# Analysis of Complex Network Attack and Defense Game Strategies Under Uncertain Value Criterion

**DOI:** 10.3390/e27101066

**Published:** 2025-10-14

**Authors:** Chaoqi Fu, Zhuoying Shi

**Affiliations:** 1 Equipment Management and UAV Engineering School, Air Force Engineering University, Xi’an 710051, China; 2 Air Defense and Antimissile School, Air Force Engineering University, Xi’an 710051, China

**Keywords:** complex networks, games, value evaluation criteria, probability inference, penalty coefficients

## Abstract

The study of attack–defense game decision making in critical infrastructure systems confronting intelligent adversaries, grounded in complex network theory, has emerged as a prominent topic in the field of network security. Most existing research centers on game-theoretic analysis under conditions of complete information and assumes that the attacker and defender share congruent criteria for evaluating target values. However, in reality, asymmetric value perception may lead to different evaluation criteria for both the offensive and defensive sides. This paper examines the game problem wherein the attacker and defender possess distinct target value evaluation criteria. The research findings reveal that both the attacker and defender have their own “advantage ranges” for value assessment, and topological heterogeneity is the reason for this phenomenon. Within their respective advantage ranges, the attacker or defender can adopt clear-cut strategies to secure optimal benefits—without needing to consider their opponents’ decisions. Outside these ranges, we explore how the attacker can leverage small-sample detection outcomes to probabilistically infer defenders’ strategies, and we further analyze the attackers’ preference strategy selections under varying acceptable security thresholds and penalty coefficients. The research results deliver more practical solutions for games involving uncertain value criteria.

## 1. Introduction

Critical infrastructure systems face persistent threats from malicious attacks, and their security has garnered widespread attention [1,2,3,4,5,6]. A prominent research direction entails leveraging complex networks to characterize the topological relationships among critical infrastructure components, coupled with game theory to analyze the strategic equilibrium between intelligent attackers and defenders under constrained offensive and defensive resources [7,8,9,10].

In complex networks, each node is an abstraction of the components in the complex system, and the edges reflect the relationships between the components. From a network perspective, all nodes and edges collectively constitute an integrated whole. Consequently, unlike traditional game models, the value assessment of each target (node) in the network is shaped not only by its intrinsic value but also by its topological relationships [11,12]. The results of networked games must account for additional payoffs beyond the direct gains from attacking or defending targets, which introduces non-linear effects into network-based attack–defense games [13,14]. To date, significant progress has been achieved in research on attack–defense games within complex networks [10,15]. Li et al. [16] studied a simultaneous-move attacker–defender game model under complete information. The strategies and payoffs in this game are defined based on the topology of the infrastructure system, and the Nash equilibrium conditions for two strategies—targeted and random—were analyzed. Li et al. [17] further investigated a Stackelberg game with complete information involving one defender (the leader) and one attacker (the follower). Their research found that, in networks with more heterogeneous distributions and fewer connections, the threshold parameters (where both players adopt random strategies) have higher values. He et al. [18] studied synchronous and sequential games in cyber-physical systems (CPS) under complete information. They used a product-form function with cyber and physical exponential correlation coefficients to characterize the effect of cyber-physical interdependency on the CPS survival probability. The results demonstrate that these correlation coefficients significantly influence the CPS survival probability. Shan et al. [19] applied game theory to model three-level defense and attack strategies in smart grid network security. They characterized the best responses and equilibrium strategies for both the attacker and defender, finding that the attacker’s optimal strategies across all three network types are unaffected by interdependent relationships between networks. In contrast, the defender’s best response depends not only on direct attacks but also on cascading effects from connected networks. Fu et al. [20] studied a two-player simultaneous game under complete information, incorporating the impact of load cascading failures on the network. By categorizing nodes into high-value and equal-value groups, they found that, if the defender’s resource allocation ratio for high-value nodes falls below a threshold, it creates an exploitable opportunity for the attacker. They also studied attack and defense games on interdependent networks, considering the cascading effects of dependencies between subnetworks [21]. Wu et al. [22] proposed a framework integrating game theory and network science, where critical target selection and resource allocation are incorporated into the strategy model. Using cumulative prospect theory, they evaluated payoffs by considering agents’ risk attitudes. Their analysis of optimal strategies under varying resource levels highlights the importance of balancing investments between enhancing component capacity and safeguarding critical components.

However, all the above studies rely on the framework of complete-information games—an assumption that deviates significantly from real-world conditions. On the one hand, the defender can deliberately conceal network information, rendering it exceedingly challenging for attackers to acquire comprehensive knowledge of the system. Nagaraja et al. [23] developed an iterative dynamic game to capture the interactions between attack and defense strategies in scale-free networks, and they provide a framework for analyzing defense and attack in networks where topology matters. Domingo et al. [24] explored iterated attack and defense in scale-free weighted and/or directed networks, and they achieved this by considering costs: there are bounds to what an attacker and a defender can do in each round. Nystrom et al. [25] consider the attacker’s state of information with respect to the defender’s network and propose a program that extends the limited attacker information state by consuming resources as part of the attack round or a probability mechanism related to network attacks. Dong et al. [26] suggest that existing methods lack consideration of fuzziness and uncertainty when evaluating the payoffs in practical attack and defense games, and they propose using intuitionistic fuzzy sets to depict such uncertain payoffs. On the other hand, the defender can also deceive attackers by means of disguise or disseminating false information, which may induce the latter to make misinformed decisions. Zeng et al. [27] proposed an active defense by revealing the disguised network to the attacker under asymmetric information, where misleading information leads to the income of the attacker being lower than expected, and the attack may even fail. Wu et al. [28] considered the risk attitudes of both the attacker and defender and studied resource allocation when the defender has multiple strategies (active strike, preventive strike, and imperfect false targets). The results show that the introduction of risk attitudes leads the game to a lose–lose situation under some circumstances and benefits one party in other cases. Zhang et al. [29] studied resource allocation among multiple targets in a defender–attacker game considering false targets, and they found that the reasonable allocation of resources for the camouflage/identification of false targets has a great impact on the final profit. Fu et al. [30] analyzed the impact of camouflage strategies on the Stackelberg game model in complex networks and found that the penalty coefficient and camouflage coefficient must be designed in combination to achieve effective defense effects. Of course, attackers may also use certain means to conceal or cover up their attack behavior, making it difficult for defenders to obtain timely or accurate network status information. The incomplete information model established by Keith et al. [31] takes into account the case in which the defender cannot distinguish or recognize the opponent’s actions and natural state. Zhang et al. [32] proposed a novel approach to the H∞ performance analysis of discrete-time networked systems subject to network-induced delays and malicious packet dropouts, aiming to identify secret attacks launched by adversaries. Hunt and Zhuang [15] conducted a comprehensive review and future development path analysis of research on offensive and defensive game problems in recent years, and they suggest that game decision making under uncertain information is a major challenge preventing theoretical research from being applied in practice.

Attack and defense game analysis is built upon two fundamental prerequisites: on one hand, it is necessary to determine the information held by the attacker and the defender separately; on the other hand, it demands an understanding of the opponent’s decision-making preferences or inclinations [33,34]. One issue that is often neglected is the evaluation of network target values—a process influenced by both subjective and objective factors. Objective factors, such as the network topology structure, are well defined, whereas subjective factors—including players’ perceptions of the target value—tend to be ambiguous. Asymmetric value perception will lead to discrepancies in the evaluation results between the attacking and defending parties. Therefore, the evaluation of the network target value is not only a factor with dual impacts but also a core element of attack and defense games: it determines the final payoffs of the game and, in turn, dictates the strategies employed by both sides. Most prior studies rely on two types of simplifying assumptions regarding target value evaluation criteria: either the criteria are uniform across attackers and defenders or both parties have complete knowledge of each other’s evaluation frameworks. However, in reality, these evaluation criteria may diverge, and one-shot games render an accurate assessment of the opponent’s target valuations exceedingly challenging. A notable finding in zero-sum games is that, when both players share identical preferences and no additional payoffs are considered, the defender incurs the maximum possible losses [28]. This creates an incentive for players to conceal their preferences from one another. Therefore, value perception asymmetry not only exists in reality but is even actively created by both offensive and defensive parties. Strictly speaking, the issue of asymmetric value perception falls under the category of incomplete information games. Unlike the incomplete grasp of objective facts—such as network information or strategic information—the uncertainty stemming from value perception asymmetry originates from the cognitive uncertainty of decision-makers. It is far more difficult and complex to collect and analyze intelligence to identify opponents’ value recognition criteria; furthermore, the conclusions derived inherently possess fuzzy attributes and subjective biases. Consequently, the study of asymmetric value recognition holds greater practical significance for decision making in network-based games.

In this paper, we study the Stackelberg game model, where the defender takes action first and the attacker takes action later. However, the attacker cannot obtain the defender’s strategic information unless the attacker takes detection actions (there is a risk of being recognized by the network). We analyze the advantageous strategy ranges and strategy transition relationships for both the attacker and the defender under distinct target value evaluation criteria. Furthermore, beyond the scope of advantageous strategies, we propose a small-sample-based Bayesian inference method to provide decision support for the attacker across varying acceptable security thresholds and penalty coefficients. Our findings offer a more practical solution for game-theoretic decision making under conditions of information uncertainty.

The article is structured as follows. In Section 2, we introduce the basic model, including network models and game models. The analysis of attack and defense strategies is shown in Section 3, where Section 3.1 analyzes the stable solution of the game under different value coefficients, Section 3.2 implements decision analysis based on probabilistic inference under uncertain conditions, and, in Section 3.3, the validation of the research results on real networks is presented. Finally, we provide conclusions in Section 4.

## 2. Basic Model

Assuming that the network model is an undirected graph G(V,E), the set of nodes is recorded as $V=\{v_{1},v_{2},\ldots ,v_{n}\}$, and the number of nodes is N=|V|; $E=\{e_{1},e_{2},\ldots ,e_{m}\}$ is the set of edges, with a total of M=|E| edges. Both the attacker and defender target network nodes for their offensive and defensive actions. The value of a node is assumed to be positively correlated with its degree centrality—defined as the number of edges directly connecting the node with other nodes in the network. The physical meaning is that nodes with higher connectivity play a more important role in the network. This implies that the higher a node’s degree, the greater its value [16]. Existing research has shown that removing nodes with high degree centrality exerts a more significant impact on the network topology and is more likely to trigger network collapse [11]. Additionally, betweenness centrality is another commonly used indicator for evaluating node importance, and ref. [35] has demonstrated the correlation between degree and betweenness. Degree has lower computational complexity than betweenness in large-scale networks.

However, there is a difference in the perception of the node value between the attacker and defender. We define the attacker’s perception of the value of node $v_{i}$ as $L_{i}^{A}=k_{i}^{\theta_{a}}$, satisfying $\theta_{a}\geq 0$. Meanwhile, the defender’s perception of the value of node $v_{i}$ is $L_{i}^{D}=k_{i}^{\theta_{d}}$, satisfying $\theta_{d}\geq 0$, where $\theta_{a}$ and $\theta_{d}$ are independent variables. The total resources of the attacker and the defender are represented as $TC_{A}$ and $TC_{D}$, respectively. The attack (defense) resources required by nodes are usually positively correlated with their value; in this paper, we define the resource consumption of attack and defense node $v_{i}$ as $c_{i}^{a}=c_{i}^{d}=k_{i}$. This is motivated by the following considerations: on the one hand, the cost of attack (defense) is only considered to be related to the objective state of the network (topology), and, on the other hand, this means that our analysis focuses only on the impact of payoffs (value criteria) on game strategies. Due to both attackers and defenders recognizing the impact of damage to high-degree nodes on the network, the attacker may not be able to successfully attack high-degree nodes. On the other hand, under the constraint of limited resources, if the defender concentrates his resources on protecting high-degree nodes, the attacker has a higher probability of succeeding in attacking the remaining nodes, which will prompt the defender to transfer defense resources from high-value nodes. The above process is a typical game process, wherein both parties must consider the strategies that their opponents may adopt. Whether a stable solution exists in this process, and how to select optimal offensive and defensive strategies, requires more effective analysis.

The attack and defense game model can be represented as

$$A:\underset{x_{i}\in S^{A},y_{i}\in S^{D}}{\max}B^{a},D:\underset{x_{i}\in S^{A},y_{i}\in S^{D}}{\max}B^{d}$$

where $S^{A}$ and $S^{D}$ are attack and defense strategies, respectively. If node $v_{i}$ is attacked (defended), then $x_{i}=1$ ($y_{i}=1$); otherwise, $x_{i}=0$ ($y_{i}=0$). The purpose of the attacker is to disrupt the network to the greatest extent possible, and the payoff of the attacker is the total value of all failed nodes in the network $B^{a}=\sum_{i\in \Omega }L_{i}^{A}$, where Ω is the set of failed nodes in the network, including nodes that have been successfully attacked and failed, as well as nodes that have detached from the main structure of the network. The goal of the defender is to minimize the value loss of the network as much as possible, and the objective function satisfies $B^{d}=-\sum_{i\in \Omega }L_{i}^{D}$. Game-theoretic analysis typically assumes that players possess a complete mutual understanding. However, the reality is that the attacker and defender may have different target evaluation criteria (due to subjective cognitive differences), their objective functions yield inconsistent evaluations under identical network damage scenarios, and changes in the relationship between risk and payoffs will lead to changes in the stable strategy combination of the game. In such cases, the strategic interaction between the attacker and defender is shaped by two core factors: their own value perceptions and their inferences regarding the opponent’s valuation criteria. This divergence can render conventional stable analysis ineffective.

## 3. Analysis of Attack and Defense Strategies

We examine two distinct attack–defense strategies: a preference strategy (PS) and random strategy (RS). The preference strategy refers to the attacker (defender) having a greater tendency to attack (defend) high-value nodes, adopting a roulette wheel selection mechanism for target screening, setting intervals for all nodes in the network based on the size of the $k_{i}^{\varphi }$-relationship of the degree values, and simulating the size of the probability distribution, where φ>0 is the preference coefficient of the defender (attacker) towards high-value nodes. Obviously, high-value nodes occupy larger probability intervals and are more likely to be selected as attack (defense) targets. The preference strategy ensures the optimal trend and maintains the randomness of the strategy. The random strategy is to randomly select all nodes with the same probability.

We have established the scale-free random network [36] with a scale satisfying N=1000, and the average degree of the network is $\langle k\rangle =2M/N\approx 6$. The investment of attack and defense resources is $TC_{A}=TC_{D}=2500$, and the preference coefficient satisfies φ=2. The above information can be obtained by the attacker through the collection and analysis of information intelligence. Therefore, both the attacker and defender are believed to have complete knowledge of the network topology but lack information about each other’s strategic choices. In this scenario, undefended nodes will fail when attacked, and defended nodes can successfully resist attacks. The following values were obtained by averaging over experiments on 50 independent networks.

### 3.1. Analysis of Game-Stable Solutions Under Different Value Coefficients

The stable solution of a game requires comprehensive consideration of the actions of both parties in the game. In the context of attack–defense interactions, attackers and defenders exhibit distinct behaviors when evaluating network targets under varying value coefficients—these evaluative behaviors not only drive their individual decisions but also ultimately dictate the game’s stable outcome. We first assume that both the attacker and defender have a consistent value coefficient—that is, $\theta_{a}=\theta_{d}=\theta$—so that we can conduct game analysis from the perspectives of both parties simultaneously.

**Figure 1:**
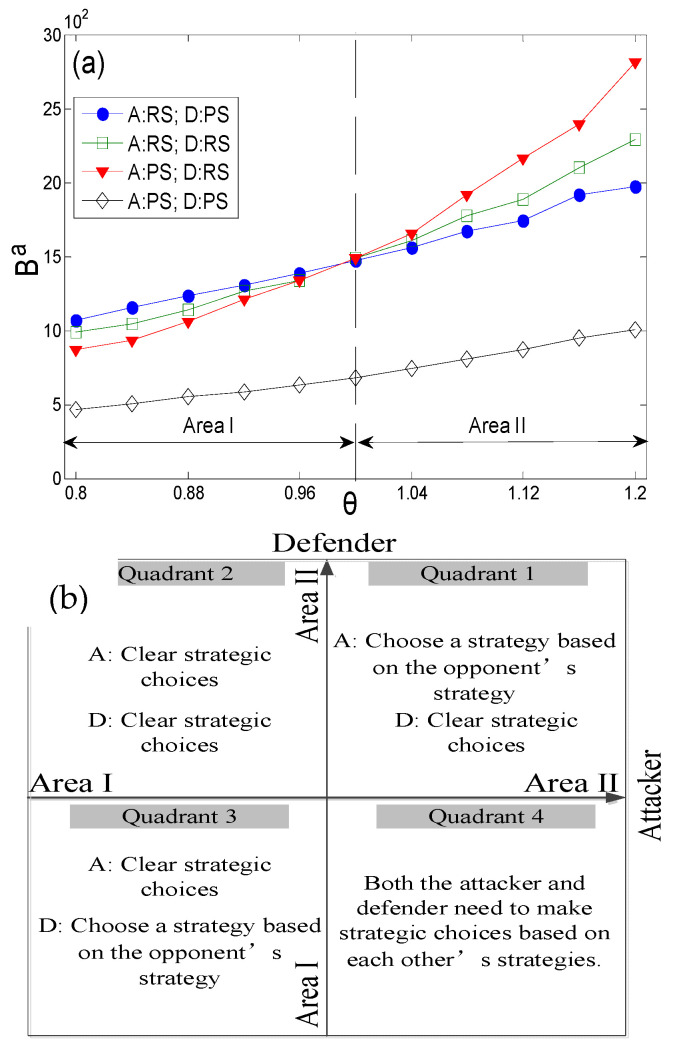
The results of attack and defense strategy combinations with different value coefficients and attack and defense decisions. ((**a**) is the result of attack and defense strategy combinations with different value coefficients; (**b**) is a schematic diagram of the area division where the attacking and defending sides make decisions).

Figure 1a shows the game results for attack and defense strategy combinations with different value coefficients. We focus on the relative relationships between different combinations of game strategies under different value coefficients, which determine the strategy choices of both parties in the game. Simulation results show that value coefficients θ have a significant impact on game results. The strategy combination of the attacker and defender is written in the form of (A,D), where A is the attacker’s strategy and D is the defender’s strategy; for example, (PS,RS) indicates that the attacker’s strategy is a preference strategy (PS), while the defender’s strategy is a random strategy (RS). According to the curve relationship, the intersection point of the curve is the critical point where the attack and defense stability strategy may change. We selected three data points as examples for game analysis, namely θ=0.8, θ=1.0, and θ=1.2, and established game matrices, as shown in Table 1, Table 2 and Table 3, for comparative analysis. The bold font in the table highlights the stable solution of the game, while the light gray background indicates the player’s advantageous strategy. Obviously, the results of game analysis vary under different value coefficients.

In practical scenarios, neither attackers nor defenders have insight into each other’s value coefficients, and their decisions are based on game matrices derived from their own value coefficients. According to the simulation results shown in Figure 1a, as the value coefficient increases, the stability of the attacker’s random strategy gradually decreases, whereas the stability of the defender’s preference strategy gradually increases. For ease of analysis, we divide the value range of coefficient θ into two regions. The significant feature of Area I is that, if the attacker’s value coefficient $\theta_{a}$ insight is in Area I, the attacker has an absolute advantage under the random strategy. Meanwhile, if the defender’s value coefficient $\theta_{d}$ insight is in Area II, the defender has an absolute advantage under the preference strategy. Figure 1b reflects the impacts of the strategies adopted by the attacker and defender across different coefficient regions. In the first quadrant, the defender can make clear decisions, whereas the attacker must base their decisions on the defender’s choices; the third quadrant presents the opposite scenario to that in the first quadrant. In the second quadrant area, both the attacker and defender will make clear decisions. Meanwhile, in the fourth quadrant, neither party can make independent decisions—instead, both need to adjust their strategies in response to the opponent’s actions.

Table 4, Table 5, Table 6 and Table 7 show the game results regarding the combination of attack and defense strategies for the defender’s value coefficient in scenarios $\theta_{d}=0.8$ and $\theta_{d}=1.12$, as well as the attacker’s value coefficient in scenarios $\theta_{a}=0.88$ and $\theta_{a}=1.2$, where $\theta_{a}=0.88$ and $\theta_{d}=0.8$ are in Area I, and $\theta_{d}=1.12$ and $\theta_{a}=1.2$ are in Area II. The stable solution in Table 4 is derived by first identifying the attacker’s advantageous strategy (random strategy), followed by the defender’s selection of the random strategy. Similarly, the stable solution in Table 7 is obtained through a sequential process: the defender first determines its dominant strategy, and the attacker then selects its own dominant strategy. In Table 6, under the corresponding value coefficients, both the attacker and the defender possess distinct advantageous strategies, and the stable solution constitutes a combination of these advantageous strategies. Table 5, by contrast, depicts a complex game scenario where neither party has an absolutely advantageous strategy. Here, the attacker and defender may make decisions based on different principles, leading to varied game outcomes.

Obviously, the final stable solution of the game needs to be determined based on the combination $\left(\theta_{a},\theta_{d}\right)$ of the value coefficient insights of the attacker and defender. The understanding of the opponent’s value coefficient by the attacker or defender is important as it can affect the final game-stable strategy. It can be confirmed that, if the value coefficient θ is small (in Area I), the attacker will adopt a random strategy, while, if the value coefficient θ is large (in Area II), the defender will adopt a preference strategy. A stable solution can be easily obtained in both of the above situations, as shown in Table 4, Table 6, and Table 7. However, in reality, it is difficult for the attacker (defender) to obtain information about the opponent’s value coefficient, especially if the attacker’s value coefficient $\theta_{a}$ insight is outside Area I and the defender’s value coefficient $\theta_{d}$ insight is outside Area II, whereby a stable strategy is difficult to obtain.

### 3.2. Decision Analysis Based on Probabilistic Inference Under Uncertain Conditions

Compared to analyzing the opponent’s value coefficient, obtaining information about the defender’s deployed actions is more readily achievable. Usually, the defender deploys a strategy earlier than the attacker, and the attacker can take detection actions to obtain information about the defender’s strategy. Therefore, the attacker has the opportunity to ascertain the defender’s strategic choice; this is beneficial in enabling the attacker to make correct decisions when the value coefficient $\theta_{a}$ insight is not in Area I. The above model is called a Stackelberg game. However, if the attacker takes action to detect the network, there is a certain probability that the network will recognize the detection action due to warning or defense mechanisms. The probability of an undefended node recognizing the detection behavior is recorded as $\omega_{1}$, and $\omega_{2}$ is the probability of a defended node recognizing the detection behavior, which usually satisfies $\omega_{2}\geq \omega_{1}$. If the network identifies the attacker’s detection action, the defense mechanism of the network is triggered. On the one hand, the attacker will no longer be able to detect the true information of the network. On the other hand, the undefended nodes will have a certain resistance ability after being attacked, and the probability of failure will be reduced from 1 to 1−β, where β denotes the penalty coefficient.

The attacker can improve the effectiveness of the attacks by detecting whether nodes are defended. However, if such detection is identified by the network, the subsequent improvement in the network’s attack resistance will adversely impact the attackers’ payoffs. Notably, large-scale detection is prone to being recognized—even under conditions where coefficients $\omega_{1}$ and $\omega_{2}$ are very low. To further maximize the benefits, the attacker can therefore first employ small-scale detection combined with probabilistic inference to deduce the defender’s strategy. This approach aims to minimize the risk of punishment caused by the detection action being identified, while maximizing the acquisition of the defender’s defense strategy. We assume that the attacker is aware of the defender’s total resources and can conduct small-scale detection tests to infer the defender’s defense strategy based on probability inference before launching a direct attack. The specific steps are as follows.

(1) The attacker first determines the acceptable security threshold $S_{\Lambda }$, which represents the level of risk that the attacker can tolerate regarding the defender identifying its detection action. $S_{\Lambda }$ will affect the sample size detected by the attacker; the larger $S_{\Lambda }$, the fewer nodes will be detected and the lower the accuracy of the attacker’s inference of the defender’s strategy based on the observed results.
(2) Nodes are randomly selected for sequential detection, with the risk of detection behavior being identified by the network denoted as $S_{T_{T}}^{T_{d}}={\left(1-\omega_{2}\right)}^{T_{d}}{\left(1-\omega_{1}\right)}^{T_{T}-T_{d}}$. $T_{T}\ll N$ is the total number of detected nodes, which means that the risk of detection behavior satisfies $S_{T}^{T_{d}}<S_{\Lambda }\;(T<T_{T})$ if and only if condition $S_{T_{T}}^{T_{d}}\geq S_{\Lambda }$ is satisfied when detecting the $T_{T}$-th. $T_{d}$ is the number of defended nodes among the detected sample. $\omega_{1}$ and $\omega_{2}$ denote the ability of the network to recognize the attacker’s detection behavior, and they do not directly affect the game results. $\omega_{1}$ and $\omega_{2}$ only affect the sampling size ($T_{d}$ and $T_{T}$) under the constraint of security threshold $S_{\Lambda }$.
(3) The attacker can calculate the benchmark ratio of defended nodes in the network using the defender’s total defense resources and publicly available/acquired network information. The proportions of nodes defended by the defender under random and preference strategies (denoted as $P_{RS}$ and $P_{PS}$, respectively) can be obtained by averaging the results from multiple simulations. The proportion of nodes under the random strategy can also be calculated through formula $P_{RS}=TC_{D}/(N\langle k\rangle )$, where $\langle k\rangle$ is the average degree of the network.
(4) Based on the benchmark ratios $P_{RS}$ and $P_{PS}$ and the sample information $T_{T}$ and $T_{d}$, the conditional probability of the defender adopting a random strategy is $P(S|RS)\approx C_{T_{T}}^{T_{d}}{\left(P_{RS}\right)}^{T_{d}}{\left(1-P_{RS}\right)}^{T_{T}-T_{d}}$. Meanwhile, the conditional probability of adopting a preference strategy is $P(S|PS)\approx C_{T_{T}}^{T_{d}}{\left(P_{PS}\right)}^{T_{d}}{\left(1-P_{PS}\right)}^{T_{T}-T_{d}}$. Here, $P(S)=T_{d}/T_{T}$ is the proportion of defended nodes in the sample, and P(RS)=P(PS)=0.5 indicates that the attacker is completely unaware of which of the two strategies the defender will employ.
(5) Based on the Bayesian formula, we can calculate the posterior values P(RS|S) and P(PS|S) using Formulas (1) and (2) and analyze the impact of estimation errors.

(1) $$P(RS|S)=\frac{P(S|RS)\times P(RS)}{P(S)}=\frac{C_{T_{T}}^{T_{d}}{\left(P_{RS}\right)}^{T_{d}}{\left(1-P_{RS}\right)}^{T_{T}-T_{d}}\times P(RS)}{\left(T_{d}/T_{T}\right)}$$

(2) $$P(PS|S)=\frac{P(S|PS)\times P(PS)}{P(S)}=\frac{C_{T_{T}}^{T_{d}}{\left(P_{PS}\right)}^{T_{d}}{\left(1-P_{PS}\right)}^{T_{T}-T_{d}}\times P(PS)}{\left(T_{d}/T_{T}\right)}$$

Obviously, as shown in Formulas (1) and (2), P(RS|S) and P(PS|S) are influenced by the prior values of $P_{PS}$, P(PS), $P_{RS}$, and P(RS), as well as the sampling results $T_{T}$ and $T_{d}$. If P(PS|S)>P(RS|S) is satisfied, it can be inferred that the defender is more likely to have adopted a preference strategy; otherwise, the defender’s strategy is deemed a random strategy. Subsequently, the attacker will select an attack strategy that is inconsistent with the defender’s strategy.

The impact of the attacker’s probability inference on their benefits is influenced by the qualitative relationship between P(RS|S) and P(PS|S), the correctness of the probability inference, and the joint influence of $S_{\Lambda }$ and β. This is usually complex and difficult to control, so we focus on analyzing the impact of $S_{\Lambda }$ and β, as these two parameters have a direct impact on the game results. The sensitivity analysis of parameters such as $\omega_{1}$, $\omega_{2}$, P(PS), and $P_{RS}$, etc., which indirectly affect the game results, can be theoretically performed based on Formulas (1) and (2).

Based on the previously set parameters, the probabilities of any node being defended under the defender’s random and preference strategies—calculated by averaging the outcomes from multiple simulations—are $P_{RS}=0.412$ (theoretical value: $P_{RS}=TC_{D}/(N\langle k\rangle )=0.417$) and $P_{PS}=0.1963$, respectively. The simulation is designed with fixed parameters $w_{1}=0.01$,$w_{2}=0.05$ and analyzes the impact of $S_{\Lambda }$ and β on the decision of the attacker under situation $\theta_{a}=1.2$. In the case of $\theta_{a}=1.2$, the attacker’s optimal choice is to adopt a strategy that is inconsistent with that of the defender. Results for other parameter value combinations can be derived using the same method.

**Figure 2:**
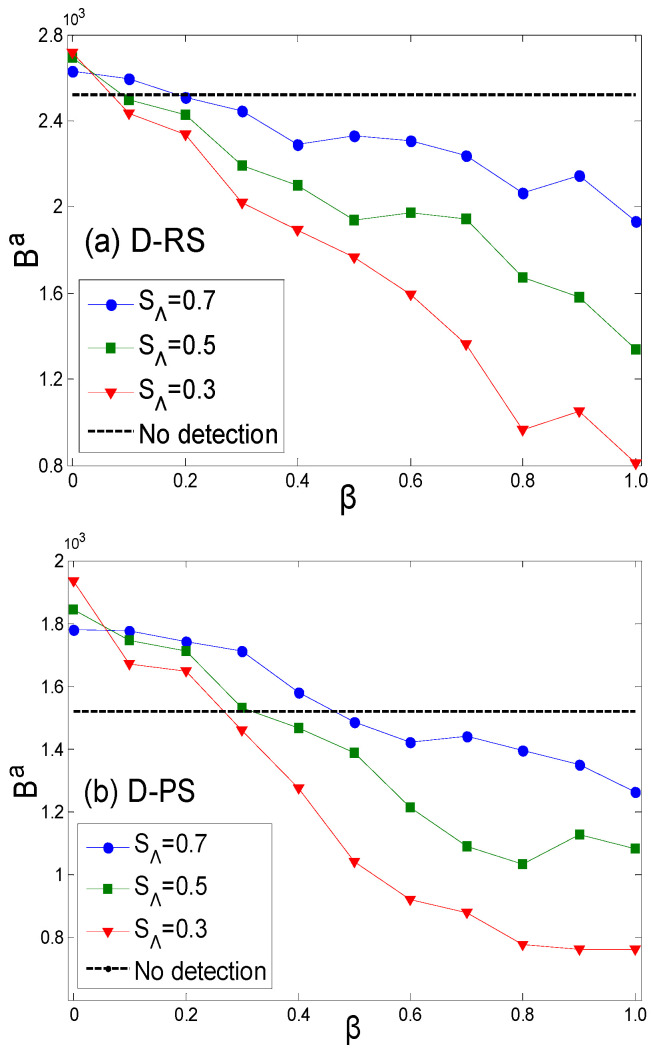
The payoff relationship of the attacker under different penalty coefficients with different acceptable security thresholds. ((**a**) and (**b**), respectively, represent the payoffs of the attacker in different scenarios $S_{\Lambda }$ under the assumption of the defender adopting an RS or PS. No detection means $S_{\Lambda }=1.0$).

Figure 2a,b illustrate the attacker’s payoffs under the assumption that the defender adopts a random strategy (RS) and a preference strategy (PS), respectively. The attacker infers the defender’s strategy from a small sample and then determines its own attack strategy. The simulation results indicate that $S_{\Lambda }$ and β indeed influence the attacker’s decision of whether to conduct probing. Under the assumption that the defender adopts a random strategy, the attacker’s detection behavior is advantageous only when the penalty coefficient β is small—in contrast to not detecting at all. However, in the case where the defender adopts a preference strategy, the attacker still has the motivation to probe the network when facing a larger penalty coefficient. Another conclusion is that the security threshold $S_{\Lambda }$ needs to be set to an appropriate value. If $S_{\Lambda }$ is too small, excessive sample detection will increase the risk of being recognized by the network, and, under the influence of the penalty coefficient, it will actually reduce the reduce the attacker’s payoffs. If it is too large, the sample size will be insufficient to accurately infer the defender’s strategy, making it difficult for the attacker to make more favorable decisions. Table 8 shows the sample sizes and defense count ranges obtained from simulations under different parameter settings.

The combined effect of $S_{\Lambda }$ and β directly impacts the results of the game. If the total value of undefended nodes among the attacked nodes is V, the expected benefit obtained by the attacker from the undefended nodes is $V*(1-S_{\Lambda })*(1-\beta )+\sigma$, where $S_{\Lambda }<1$ ($S_{\Lambda }=1$ means no detection), and $1-S_{\Lambda }$ denotes the probability of the attacker’s detection behavior being identified by the network. If the network fails to recognize the attacker’s detection behavior, then β≡0; otherwise, β is the corresponding value. σ is the value of nodes separated from network entities. Meanwhile, the inference results of the attacker regarding defense strategies can also affect V; correct inferences will increase V, while incorrect inferences will decrease the value of V. Moreover, the inference result will be affected by $S_{\Lambda }$. Therefore, the sensitivity of $S_{\Lambda }$ and β to the game result is intricate.

**Figure 3:**
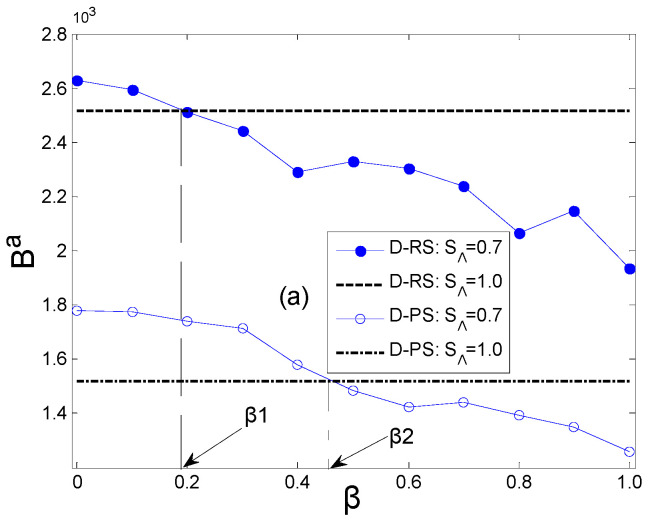

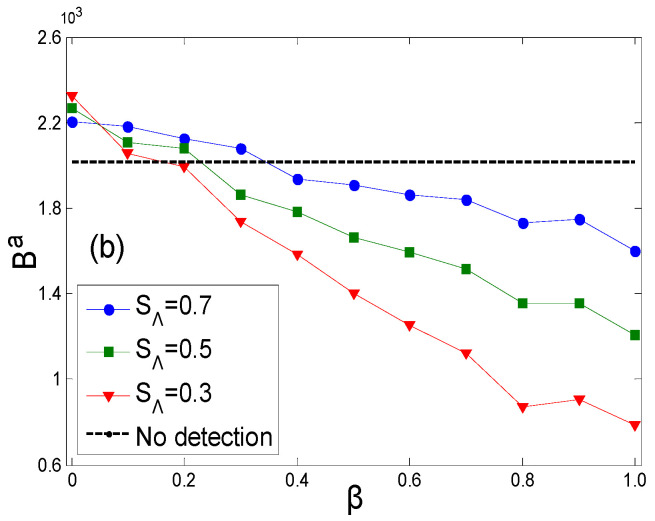
The payoff relationship of the attacker under different penalty coefficients. ((**a**) demonstrates the difference in penalty coefficient β for the attacker carrying out detection behavior under different strategies of the defender at security threshold $S_{\Lambda }=0.7$; (**b**) showcases the impacts of detection behavior and penalty coefficient β on the payoffs of the attacker under different security thresholds $S_{\Lambda }$).

However, in reality, the attacker lacks clarity on whether the defender has adopted a preference strategy (PS) or random strategy (RS). Therefore, the attacker cannot definitively decide whether to conduct detection under a given security threshold. As shown in Figure 3a, when the defender adopts a PS or RS, respectively, the penalty coefficient corresponding to whether the attacker conducts detection behavior under the security threshold $S_{\Lambda }=0.7$ is different. Obviously, if $\beta <\beta_{1}$, the attacker should conduct detection behavior, and, if $\beta >\beta_{2}$, no detection behavior should be performed. Under the condition of $\beta_{1}<\beta <\beta_{2}$, the attacker cannot decide whether to detect or not. Therefore, the aspect that the attacker needs to analyze is the strategic advantage of different security thresholds and penalty coefficients when the defender’s strategy (PS or RS) is unknown. Figure 3b is the result of the comprehensive processing of the data in Figure 2a,b, indicating the impacts of decisions made by the defender with equal probability regarding the attacker’s payoffs under the two strategies (PS or RS). According to the results in Figure 3b, the attacker can determine whether to conduct detection behavior based on different penalty coefficients for the defender at different security thresholds.

### 3.3. Verification and Analysis of Real Networks

To verify the effectiveness of the proposed method, we conducted simulation verification on two real networks—the Email network [20] and the US airlines network [37]. The relevant network parameters are shown in Table 9.

**Figure 4:**
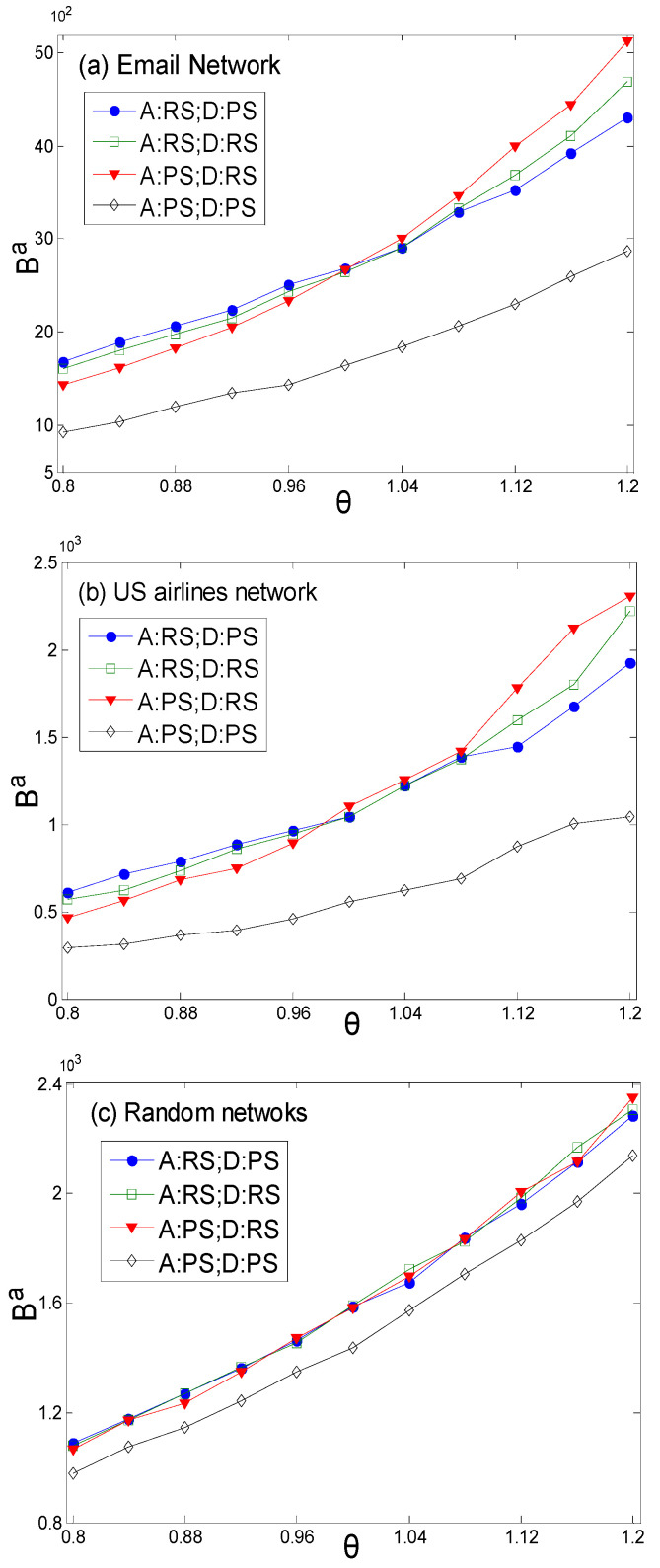
The results of attack and defense strategy combinations with different value coefficients. (**a**,**b**) are the real networks—the Email network and US airlines network, respectively—and (**c**) is the comparative network—a random network.

**Figure 5:**
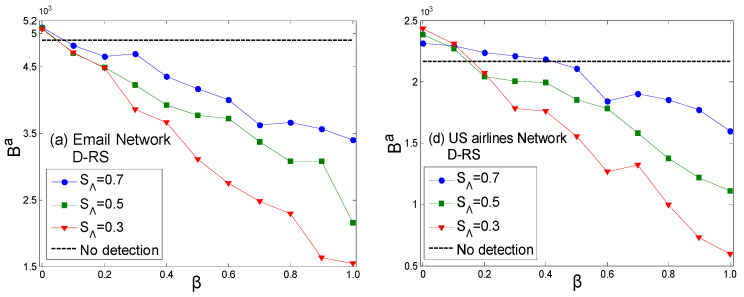

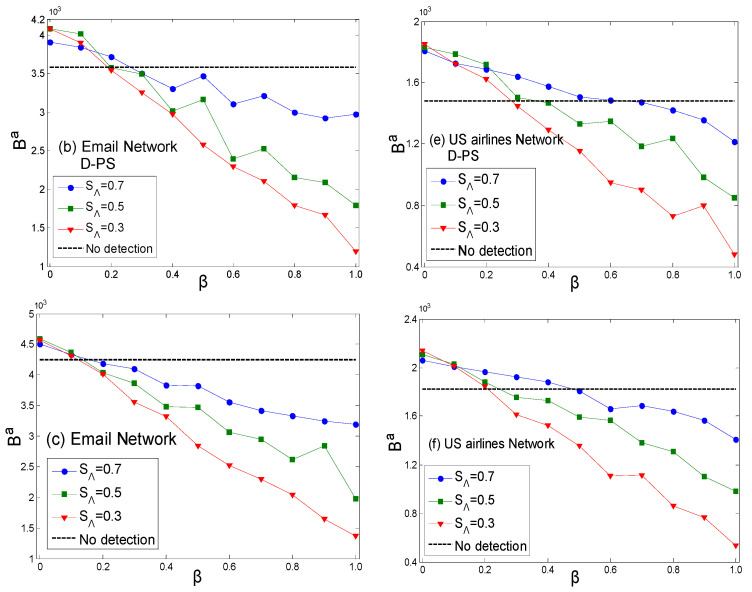
The payoff relationship of the attacker under different penalty coefficients with different acceptable security thresholds. ((**a**)\(**d**) and (**b**)\(**e**), respectively, represent the payoffs of the attacker in different scenarios $S_{\Lambda }$ under the assumption of the defender adopting an RS or PS. (**c**)\(**f**) show the payoffs of the attacker in different scenarios without considering the strategy of the defender. Among them, (**a**–**c**) are the calculation results for the Email network, and (**d**–**f**) are the calculation results for the US airlines network).

The simulation results in Figure 4a,b have a high degree of similarity with Figure 1a, as the topologies of the Email network and US airlines network are similar to that of the scale-free network, all exhibiting obvious power-law distribution characteristics. Therefore, node value evaluation based on the topological structure yields similar relative results under the combination of preference and random strategies. Figure 4c shows the calculation results for the random network [38]. The topology of the random network follows a normal distribution, and most nodes have similar probability distributions in the roulette wheel of the preference strategy. Therefore, the similarity between the node sets of the attack and defense sides under the preference strategy is lower than that of the scale-free distribution network, and it is closer to the screening results of the random strategy. This is the reason that there is little difference in the results of strategy combinations (RS,RS), (RS,PS), (PS,RS). This indicates that the heterogeneity of the network topology is the root cause of the attacker and defender having ranges of advantageous value coefficients in the game under different strategy combinations. Figure 5 shows the payoffs of the attacker in different scenarios. According to Figure 5c,f, this can provide a reference for the attacker to determine whether to carry out detection behavior under different combinations of security thresholds and penalty coefficients.

## 4. Conclusions

Critical infrastructure systems are complex systems composed of numerous interconnected components. Complex networks can effectively model the topological relationships between these components. The value of a target (node) in the network depends not only on its intrinsic monetary value but also on its structural position and functional role, as reflected by the network’s topology. However, the attacker and defender may assess the target value differently, leading to different decisions in strategic interactions.

In this paper, we simulate and analyze how differing target value assessments between the attacker and defender influence the game dynamics. Our findings reveal that both sides have distinct advantage ranges in their value coefficients. Within these advantage ranges, a player can make optimal decisions without considering the opponent’s valuation. Outside these ranges, we investigate how the attacker uses Bayesian inference on small-sample observations to deduce the defender’s strategy. The results demonstrate that (1) the defender’s penalty coefficient (measures taken by the defender after discovering the attacker’s detection behavior) and the attacker’s acceptable security threshold (risk of the attacker’s detecting behavior being recognized by the defender) significantly influence whether the attacker conducts detection actions; (2) with information about the penalty coefficient and the total defense resources, the attacker can decide whether to perform small-sample sampling, and they can select an appropriate security threshold to increase the payoffs.

This study provides a more practical approach for games under information uncertainty in undirected networks. In fact, our model and method can be applied to weighted networks by replacing node degrees with node weights. However, securing critical infrastructure against intelligent adversaries remains an open challenge. Target valuation needs to account for multiple factors beyond the topology (e.g., functional attributes, group dependencies). Additionally, game-theoretic analysis typically assumes that players understand their opponents’ information, which is often not the case in practice. Future research should focus on reducing the reliance on opponent information or substituting hard-to-obtain data with more accessible alternatives.

## Figures and Tables

**Table 1:** The payoff matrix of an attack and defense game (θ=0.8).

**Attacker**		**Defender**	
		Random strategy	Preference strategy
	Random strategy	**985.6, −985.6**	1064.7, −1064.7
	Preference strategy	869.3, −869.3	465.1, −465.1

**Table 2:** The payoff matrix of an attack and defense game (θ=1.0).

**Attacker**		**Defender**	
		Random strategy	Preference strategy
	Random strategy	1429.1, −1429.1	1470.4, −1470.4
	Preference strategy	1465.9, −1465.9	668.6, −668.6

**Table 3:** The payoff matrix of an attack and defense game (θ=1.2).

**Attacker**		**Defender**	
		Random strategy	Preference strategy
	Random strategy	2292.2, −2292.2	**1971.7, −1971.7**
	Preference strategy	2815.5, −2815.5	1005.8, −1005.8

**Table 4:** The payoff matrix of an attack and defense game ($\theta_{a}=0.88,\theta_{d}=0.8$).

**Attacker**		**Defender**	
		Random strategy	Preference strategy
	Random strategy	**1142.8, −985.6**	1233.3, −1064.7
	Preference strategy	1062, −869.3	549.4, −465.1

**Table 5:** The payoff matrix of an attack and defense game ($\theta_{a}=1.2,\theta_{d}=0.8$).

**Attacker**		**Defender**	
		Random strategy	Preference strategy
	Random strategy	2292.2, −985.6	1971.7, −1064.7
	Preference strategy	2815.5, −869.3	1005.8, −465.1

**Table 6:** The payoff matrix of an attack and defense game ($\theta_{a}=0.88,\theta_{d}=1.12$).

**Attacker**		**Defender**	
		Random strategy	Preference strategy
	Random strategy	1142.8, −1887.5	**1233.3, −1744.3**
	Preference strategy	1062, −2162.5	549.4, −867.7

**Table 7:** The payoff matrix of an attack and defense game ($\theta_{a}=1.2,\theta_{d}=1.12$).

**Attacker**		**Defender**	
		Random strategy	Preference strategy
	Random strategy	2292.2, −1887.5	**1971.7, −1744.3**
	Preference strategy	2815.5, −2162.5	1005.8, −867.7

**Table 8:** Sample sizes and defense count ranges obtained from simulations.

Type of Network	RS/PS	S_∧_	Sample Size (*T_T_*)	Defended Count (*T_d_*)
Scale-free network	RS	0.7	12.04	1.695
		0.5	19.17	2.755
		0.3	26.94	3.535
	PS	0.7	17.83	2.614
		0.5	28.37	3.141
		0.3	38.8	3.88

**Table 9:** Network parameter settings.

Network Type	*N*	<*k*>	*TC_A_* and *TC_D_*	*P_RS_*	*P_PS_*
Email Network	1133	9.62	4500	0.4038	0.1832
US Airlines Network	332	12.81	1800	0.38	0.102
Random Network	1000	6.62	2700	0.4038	0.377

## Data Availability

The original data presented in the study are openly available in Network Repository at reference [37].
